# *Proteus*-species-associated periprosthetic hip and knee joint infections – a 15-year cohort analysis

**DOI:** 10.5194/jbji-10-265-2025

**Published:** 2025-08-11

**Authors:** Veronika Achatz, Jennyfer A. Mitterer, Stephanie Huber, Ece Akcicek, Selma Tobudic, Sujeesh Sebastian, Jochen G. Hofstaetter

**Affiliations:** 1 Michael Ogon Laboratory for Orthopaedic Research, Orthopaedic Hospital Speising, Vienna, Austria; 2 AUVA Trauma Center Vienna Meidling, Vienna, Austria; 3 2nd Orthopaedic Department, Orthopaedic Hospital Speising, Vienna, Austria; 4 Department of Medicine I, Division of Infectious Diseases and Tropical Medicine, Medical University of Vienna, Vienna, Austria

## Abstract

**Introduction**: While Gram-negative periprosthetic joint infections (PJIs) are generally known for their poor outcome, few data on *Proteus* species exist. Therefore, we investigated the prevalence, clinical characteristics, microbial spectrum, outcomes, antimicrobial treatment, and surgical procedures of *Proteus*-species-associated PJIs. **Methods**: We retrospectively evaluated 1776 culture-positive revision hip and knee arthroplasties (hereafter rTHA and rTKA, respectively) from a single institution between 2008 and 2024. The European Bone and Joint Infection Society and International Consensus Meeting criteria were used for classification. The Charlson comorbidity score and tier classification were used for evaluating risk factors and success and failure rates. Statistical analysis was performed using the chi-square test and binary logistic regression. **Results**: Among 1776 culture-positive revision arthroplasties, we identified 26 (1.5 %) *Proteus*-species-associated PJIs. The majority were observed in rTHA, mostly in chronic (65.4 %) and polymicrobial (57.7 %) infections. Chronic PJIs were associated with polymicrobial infections ( p=0.027 ), resulting in a higher failure rate ( p=0.041 ). Among polymicrobial infections (15 of 26 cases), *Enterococcus faecalis* (5 of 15), *Staphylococcus epidermidis* (4 of 15), and *Pseudomonas aeruginosa* (3 of 15) were most frequently observed. The most frequently used surgical approach was a two-stage revision (46.2 %), with a success rate of 25 % (3 of 12). *Proteus*-species-associated PJIs were mainly treated with fluoroquinolone, especially ciprofloxacin showed higher success rates ( p=0.018 ). The reinfection-free survival rate was 48.5 % after 12 months and 22.6 % after 40 months. **Conclusion**: *Proteus* species represent a rare group of pathogens and are predominantly found in chronic and polymicrobial PJIs, with a higher occurrence in rTHA than rTKA. Despite an overall high clinical failure rate, ciprofloxacin showed promising antimicrobial treatment efficacy.

## 1 Introduction

Periprosthetic joint infections (PJIs) are among the most severe complications of total joint arthroplasty (TJA). While PJIs are predominantly caused by Gram-positive (GP) pathogens, Gram-negative (GN) bacteria are increasingly being reported as causing PJIs (up to 23 % of cases) (Benito et al., 2016; Hsieh et al., 2009; Sebastian et al., 2019). Although GN PJIs are associated with poor outcomes, most studies have focused on GP pathogens; thus, data on GN-pathogen-associated PJIs remain limited (Aboltins et al., 2011; Tande and Patel, 2014; Uçkay and Bernard, 2010).

*Pseudomonas* (20 %–36 %) and *E. coli* (3 %–30 %) are the most common pathogens among GN PJI, whereas *Proteus *spp. only comprise 3 %–15 % of cases (Hsieh et al., 2009; Rodríguez-Pardo et al., 2014; Zmistowski et al., 2011). *Proteus* spp. are GN opportunistic pathogens known for their clinical manifestation in urinary tract infections (UTIs) and catheter-associated urinary tract infections (CAUTIs) (Schaffer and Pearson, 2015). While recent data indicate a growing number of infections caused by *Proteus*, little is known about *Proteus*-species-associated PJI (Armbruster et al., 2018; Facciolà et al., 2022).

Recent studies have proposed that outcomes and treatments differ greatly among GN bacteria, necessitating pathogen-specific therapies (Gonzalez et al., 2024; Uçkay and Bernard, 2010). Given the increasing incidence of GN PJIs and their unique challenges, pathogen-specific studies are required to better understand and manage infections caused by GN organisms. Additionally, the growing concern of antimicrobial resistance among *Proteus* isolates further complicates the management of these infections and may necessitate extended antimicrobial therapy and multiple surgical interventions.

Therefore, this study evaluated the frequency, clinical characteristics, microbial spectrum, surgical procedures, antibiotic treatment, and outcome of total hip and knee arthroplasty revisions (hereafter rTHA and rTKA, respectively) associated with *Proteus* spp.

## 2 Materials and methods

After receiving institutional ethics board approval (EK 10/2020; blinded for review), we investigated 1776 culture-positive knee and hip revision arthroplasties from January 2008 until June 2024 from our prospectively maintained in-house arthroplasty registry and PJI database. All patients who had a positive intraoperative culture for *Proteus* spp. were included in this study. For PJI classification, the International Consensus Meeting (ICM) 2018 (Shohat et al., 2019) and the European Bone and Joint Infection Society (EBJIS; McNally et al., 2021) criteria were used. PJI cases were classified as acute if the onset occurred within 3 months or as chronic if the onset was longer than 3 months after the primary implantation (Li et al., 2018). Patient specific risk factors were assessed, using the McPherson classification (Coughlan and Taylor, 2020) and the Charlson comorbidity index (CCI; Charlson et al., 1987).

### 2.1 Microbiological analysis

For microbiological analysis, the following samples were utilized: preoperatively collected synovial fluid, periprosthetic tissue samples, intraoperative swabs, and sonication fluid. Swabs were accepted as positive cultures only when collected intraoperatively. The median number of samples taken per surgery was five, with a median number of four positive intraoperative cultures. Explanted devices were immediately placed into sonication containers, into which saline solution was added to completely cover the implants. The container was then sonicated and vortexed (Trampuz et al., 2007). Tissue samples and sonication fluid (0.1 mL) were further analysed for bacterial and fungal identification using standard microbiological techniques (Frank et al., 2021). Antimicrobial susceptibility profiling was determined using the BD system (Becton Dickinson and Company, Franklin Lakes, NJ), according to the manufacturer's recommendations, and interpreted following the European Committee on Antimicrobial Susceptibility Testing (EUCAST) guidelines (EUCAST, 2024).

Antibiotic treatment was initially given according to the institutional protocols and subsequently adjusted based on the antibiogram results. Antibiotic resistance was evaluated in all patients. All patients received antimicrobial treatment consisting of at least two antimicrobial agents in combination.

### 2.2 Follow-up and clinical outcome

Four patients died during the follow-up period. Patients who had treatment failure within 1 year were included, although they did not achieve a 1-year follow-up. If additional information on the follow-up was needed, patients were contacted by phone. Detailed demographic data are displayed in Table 1.

The treatment failure and success rates were calculated using the tier classification by the Musculoskeletal Infection Society (Fillingham et al., 2019). Cases classified in the first group of the tier classification (Tier 1) were considered successful, as the infection was successfully eradicated without further antibiotic treatment. We also included spacer implantation followed by successful reimplantation and complete infection control in the first category. Tier 2 includes patients on suppressive antibiotic therapy. Tier 3 consists of all patients with following surgery and is divided into different subcategories: A – aseptic revision after 1 year; B – septic revision after 1 year; C – aseptic revision within 1 year; D – septic revision within 1 year; E – amputation, resection arthroplasty, and arthrodesis; F – retained spacer. Tier 4 includes all of the patients who either died within 1 year (A) or after 1 year (B).

### 2.3 Statistical analysis

Demographic variables are presented as means with standard deviations. The body mass index (BMI) and CCI are shown as the mean, standard deviation, and interquartile range (IQR) for age, calculated for all patients. Categorical variables were compared using the chi-square test, while continuous variables were analysed using the Mann–Whitney U test or the t test for normally distributed values. Results were accepted as statistically significant at a p value < 0.05. Statistical analyses were performed using IBM SPSS Statistics version 26 (SPSS Inc, Chicago, IL, USA).

**Table 1:** Demographic data of the study population.

		Culture-positive revision TJA (2008–2024)	
		Proc. no.: 1776	
		Hip/knee: 1036/740	
		Pat. no.: 1389	
		*Proteus*-species-positive revision TJA	
		Proc. no. (%): 26/1776 (1.5)	
		Pat. no. (%): 25/1389 (1.8)	
	Total Proc.: 26 Pat.: 25	Hip Proc.: 23/26 (88.5 %) Pat.: 22/25 (88.0 %)	Knee Proc.: 3/26 (11.5 %) Pat.: 3/25 (12.0 %)
Male/female	8/17	6/16	2/1
Age in years (mean ± SD)	71.2 ± 12.2	71 ± 12.8	74 ± 5.3
BMI (mean ± SD)	32.0 ± 7.3	32.6 ± 7.5	28.2 ± 3.5
UPIC	1 (3.8)	1 (3.8)	0 (0.0)
Septic	25 (96.2)	22 (84.6)	3 (11.5)
Acute PJI (%)	9 (36)	7 (28)	2 (8)
Chronic PJI (%)	16 (64)	15 (60)	1 (4)
*Inf. primary TJA (%)*	*9/25 (36)*	*8 (32)*	*1 (4)*
*Inf. revision TJA (%)*	*16/25 (64)*	*14 (56)*	*2 (8)*
Monomicrobial (%)	11 (42.3)	10 (38.5)	1 (3.8)
Polymicrobial (%)	15 (57.7)	13 (50.0)	2 (7.7)
Single-stage (%)	3 (11.5); s/f: 1/3	3 (11.5)	0
DAIR (%)	10 (38.5); s/f: 2/10	8 (30.8)	2 (7.7)
Two-stage (%)	12 (46.2); s/f: 3/12	11 (42.3)	1 (3.8)
UPIC (%)	1 (3.8); s/f: 0/1	1 (3.8)	0
ICM 2018			
Infected (%)	24 (92.3)	21 (80.8)	3 (11.5)
Inconclusive (%)	0 (0.0)	0 (0.0)	0 (0.0)
Not Infected (%)	2 (8.0)	2 (7.7)	0 (0.0)
EBJIS 2021			
Confirmed (%)	22 (84.6)	19 (73.1)	3(11.5)
Likely (%)	4 (15.4)	4 (15.4)	0 (0.0)
Unlikely (%)	0 (0.0)	0 (0.0)	0 (0.0)
CCI, median ± SD	4.3±2.1	4.1±2.1	5.7±2.1
McPherson score			
Infection grade			
I	7	7	0
II	3	1	2
III	16	15	1
Systemic host grade			
A	13	12	1
B	11	10	1
C	2	1	1
Local extremity grade			
1	4	4	0
2	18	16	2
3	4	2	2

Abbreviations/acronyms used in the table are as follows: TJA – total joint arthroplasty; SD – standard deviation; Proc. no. – procedure number; Pat. no. – patient number; Inf. – infected; UPIC – unexpected positive intraoperative culture; PJI – periprosthetic joint infection; ICM – International Consensus Meeting; EBJIS – European Bone and Joint Infection Society; CCI – Charlson comorbidity index; DAIR – debridement antibiotic, and implant retention; s/f – success/failure. Note: one patient received different surgeries consecutively, counted as one patient in DAIR and one in two-stage procedures.

## 3 Results

Out of 1776 culture-positive revision arthroplasties, 26 of 1776 (1.5 %) revision surgeries showed intraoperatively positive results for *Proteus* spp. These involved 25 patients (8 male and 17 female) who underwent hip (23 of 26, 88.5 %) and knee (3 of 26, 11.5 %) revision surgeries. One case (1 of 26, 3.8 %) was a presumed aseptic rTHA with an unexpectedly positive intraoperative culture (UPIC). Using the ICM criteria, 24 cases were defined as infections, and 2 were identified as not infected, whereas according to EBJIS criteria, 22 cases were identified as infections, and 4 were considered likely to have an infection. The mean age was 71.2 years (IQR: 78.8–68), and the mean BMI (kg m^−2^) was 32.0 ± 7.3. No statistically significant difference between BMI, CCI, age, and sex between rTHA and rTKA was found. The median follow-up period was 26.9 months (IQR: 5.8–40.6).

### 3.1 Microbial analysis and polymicrobial infections

In total, 132 samples were collected from 26 revision procedures; of these 104 (78.8 %) had a positive intraoperative culture. Overall, a total of 143 microorganisms were identified, including 80 (55.9 %) cases of *Proteus* spp. and 63 (44.1 %) cases of other pathogens. Monomicrobial infections with *Proteus mirabilis* were identified in 11 (42.3 %) of the 26 surgeries. Polymicrobial infections were found in 15 (57.7 %) of the 26 procedures, with *P. mirabilis* identified in 14 (93.3 %) cases and *Proteus vulgaris* in 1 (6.7 %) case. Moreover, polymicrobial infections were more frequently observed in patients with chronic PJI ( p=0.027 ), especially those with chronic hip PJI ( p=0.02 ). The same effect could not be shown in knee PJI ( p=0.667 ). Distributions of the microbiological spectrum in rTKA and rTHA are displayed in Table 2.

**Table 2:** Detailed microbiological spectrum in PJI associated with *Proteus* spp. and categorized in hip and knee infections.

	Overall	Hip		Knee	
		Primary PJI	Revision PJI	Primary PJI	Revision PJI
Samples total, n (%)	132 (100)	27 (29.5)	82 (62.1)	7 (5.3)	16 (12.1)
Periprosthetic tissues	71 (53.8)	12 (16.9)	39 (54.9)	6 (8.5)	14 (19.7)
Intraoperative swabs	45 (34.1)	8 (17.8)	37 (82.2)	0 (0.0)	0 (0.0)
Sonication fluids	16 (12.1)	7 (43.8)	6 (37.5)	1 (6.3)	2 (12.5)
Positive microbiological samples, n (%)	104 (78.8)	25 (24.0)	57 (54.8)	7 (6.7)	15 (14.4)
Detected microorganism, n (%)	143 (100)	33 (23.1)	71 (49.7)	14 (9.8)	25 (17.5)
In monomicrobial infections, n (%)	37 (25.9)	2 (5.4)	28 (75.7)	0 (0.0)	7 (18.9)
In polymicrobial infections, n (%)	106 (74.1)	31 (29.2)	43 (41.1)	14 (13.2)	18 (17.0)
In periprosthetic tissues	74 (51.7)	12 (16.2)	30 (40.5)	12 (16.2)	20 (27.0)
In intraoperative swabs	44 (30.8)	10 (22.7)	34 (77.3)	0 (0.0)	0 (0.0)
In sonication fluids	25 (17.5)	11 (44)	7 (28)	2 (8)	5 (20)
*Co-pathogens in polymicrobial infections*	63 (44.1)	17 (27.0)	23 (36.5)	7 (11.1)	16 (25.4)
*Enterococcus faecalis*	21 (33.3)	6 (21.6)	8 (38.1)	0 (0.0)	7 (33.3)
*Pseudomonas aeruginosa*	9 (14.3)		9 (100)		
*Citrobacter koseri*	9 (14.3)		1 (11.1)		8 (50)
*Staphylococcus aureus*	7 (11.1)			7 (100)	
*Staphylococcus lugdunensis*	6 (9.5)	6 (100)			
*Staphylococcus epidermidis*	5 (9.4)	4 (80)	1 (20)		
*Escherichia coli*	4 (6.3)	4 (100)			
*Cutibacterium avidum*	1 (1.6)	1 (100)			
*Klebsiella pneumoniae*	1 (1.6)				1 (100)

The most common combinations were between *Proteus* spp. and *Enterococcus faecalis* in 5 of 15 cases (33.3 %), *Staphylococcus epidermidis* in 4 of 15 cases (26.7 %), and *Pseudomonas aeruginosa* in 3 of 15 cases (20 %). The total number of microbial samples ( n=63 ) detected in polymicrobial infections and their distribution in rTHA and rTKA are shown in Table 2. There were combinations of *Proteus* spp. with up to four microorganisms taken from one joint. The detailed combinations of pathogens in polymicrobial infections are shown in Table 3.

**Table 3:** Detailed microbial profiles, surgical details, antibiotic therapy against *Proteus* spp., and success/failure rate as per the tier classification. Every line represents one patient. Patients 23 and 24 refer to the same patient but are mentioned separately because they underwent different surgeries and treatments. Antibiotic agents targeting co-pathogens are not mentioned in this table. Please see the Supplement (Table S1) for additional details on antibiotic therapies in polymicrobial infections.

Pat. no.	Joint	Surgical procedure	Microorganisms	Prior infections	Initial antibiotic therapy	Targeted antibiotic therapy	Targeted therapy initiation (PoD)	Post targeted AB^d^	Duration (weeks)	Tier class.	Success/failure
1	rTHA	Two-stage	*P. mirabilis*	*Unknown*	Cefuroxime^a^ + moxifloxacin^a^	PIT	2	PIT, cefalexin, AMC, TRS	12	3D	failure
2	rTHA	Two-stage	*P. mirabilis* + *S. epidermidis*	*P. mirabilis*	Teicoplanin^b^ + moxifloxacin^b^	Ceftriaxone + ciprofloxacin	4	Ceftriaxone + ciprofloxacin, TRS + ciprofloxacin	7	4A	failure
3	rTHA	One-stage	*P. mirabilis* + *P. aeruginosa*	*Culture negative*	Cefuroxime^a^	Meropenem	2	Meropenem, ciprofloxacin	8	1	success
4	rTHA	DAIR	*P. mirabilis*	–	Teicoplanin^c^	Ceftriaxone	11	Ceftriaxone, cefalexin + moxifloxacin	8	3D	failure
5	rTHA	DAIR	*P. mirabilis* + *S. lugdunensis* + *S. epidermidis*	–	Teicoplanin^b^	Ciprofloxacin	7	Ciprofloxacin	9	3B	failure
6	rTHA	Two-stage	*P. mirabilis* + *P. aeruginosa*	*S. aureus*	Teicoplanin^c^ + meropenem^c^	Meropenem	0	Meropenem, ciprofloxacin	6	1	success
7	rTHA	DAIR	*P. mirabilis* + *S. lugdunensis*	–	Doxycycline^a^ + moxifloxacin^b^	TRS	8	TRS	6	1	success
8	rTHA	DAIR	*P. mirabilis*	*S. hominis*	Teicoplanin^b^ + rifampicin^b^ + doxycycline^b^	Ciprofloxacin	3	Ciprofloxacin	12	3B	failure
9	rTHA	Two-stage	*P. mirabilis* + *S. epidermidis* + *C. avidum*	–	Cefuroxime^c^ + doxycycline^a^	Aztreonam	5	Aztreonam, moxifloxacin	8	3D	failure
10	rTHA	DAIR	*P. mirabilis*	*S. epidermidis*	Doxycycline^b^ + rifampicin^b^ + daptomycin^b^	Ceftriaxone	5	Ceftriaxone, cefuroxime	8	3B	failure
11	rTKA	DAIR	*P. mirabilis*	*Unknown*	Moxifloxacin^c^ + ceftriaxone^c^	TRS + ciprofloxacin	13	TRS + ciprofloxacin	30	1	success
12	rTHA	Two-stage	*P. mirabilis*	*E. faecalis*	Cefuroxime^c^ + moxifloxacin^c^	Cefuroxime + moxifloxacin	0	Cefuroxime + moxifloxacin, cefalexin	26	3D	failure
13	rTHA	Two-stage	*P. mirabilis*	*P. mirabilis*	Teicoplanin^a^	Ceftriaxone + ciprofloxacin	1	Ceftriaxone + ciprofloxacin, ciprofloxacin + TRS	22	1	success

**Table 3:** Continued.

Pat. no.	Joint	Surgical procedure	Microorganisms	Prior infections	Initial antibiotic therapy	Targeted antibiotic therapy	Targeted therapy initiation (PoD)	Post targeted AB^d^	Duration (weeks)	Tier class.	Success/failure
14	rTHA	Two-stage	*P. mirabilis*	*E. faecalis*	Teicoplanin^b^ + moxifloxacin^b^	Moxifloxacin	0	Meropenem + moxifloxacin, cefalexin	16	3D	failure
15	rTHA	Two-stage	*P. mirabilis*	*Culture negative*	PIT^b^	Cefotaxime	3	Cefotaxime, levofloxacin	16	3F	failure
16	rTHA	Two stage	*P. mirabilis* + *E. faecalis*	*Unknown*	Cefuroxime^c^ + moxifloxacin^c^	Moxifloxacin	0	Moxifloxacin + AMC	12	3F	failure
17	rTKA	DAIR	*P. mirabilis* + *S. aureus*	–	Clindamycin^c^	Meropenem	6	Meropenem, ciprofloxacin	12	3D	failure
18	rTHA	One-stage	*P. mirabilis* + *S. epidermidis*	–	Cefuroxime^b^ + doxycycline^a^	Cefalexin	11	Cefalexin, TRS	12	4A	failure
19	rTHA	One-stage	*P. mirabilis* + *E. faecalis* + *E. coli*	–	Cefazolin^c^	Cefotaxime	7	Cefotaxime, aztreonam, meropenem ceftazidime/avibactam + fosfomycin ceftazidime/avibactam + levofloxacin	30	3D	failure
20	rTHA	Two stage	*P. mirabilis* + *E. faecalis*	–	PIT^c^	Meropenem + ciprofloxacin	Unknown	Meropenem, ciprofloxacin	24	1	success
21	rTHA	UPIC	*P. mirabilis*	–	Cefazolin^c^	Meropenem	6	Meropenem, AMC	24	3D	failure
22	rTHA	DAIR	*P. vulgaris* + *E. faecalis*	*Culture negative*	Cefazolin^b^	PIT	7	PIT, moxifloxacin, AMC, TRS, levofloxacin	17	4A	failure
23	rTHA	Two-stage	*P. mirabilis* + *E. coli*	*S. epidermidis*	Doxycycline^b^ + teicoplanin^b^	Trimethoprim	4	Trimethoprim, meropenem	12	4A	failure
24	rTHA	DAIR	*P. mirabilis*	*P. mirabilis* + *E. coli*	Doxycycline^b^ + teicoplanin^b^	Meropenem	0	Meropenem	12	4A	failure
25	rTKA	Two-stage	*P. mirabilis* + *E. faecalis* + *K. pneumoniae* + *C. koseri*	*Unknown*	PIT^c^ + cefazolin^a^	PIT	0	PIT, aztreonam, ceftazidime, ciprofloxacin	12	3D	failure
26	rTHA	DAIR	*P. mirabilis* + *P. aeruginosa* + *C. koseri* + *E. coli*	*C. glabrata* + *S. epidermidis*	Cefuroxime^a^*	Meropenem	0	Meropenem	8	3E	failure

^a^ Antibiotic therapy was given as perioperative prophylaxis. ^b^ Antibiotic therapy started before surgery and continued during surgery. ^c^ Empirical antibiotic therapy, which was started during surgery and changed accordingly to the antibiogram. ^d^ Antibiotic changes classified as continued, switched, de-/escalated, or salvage therapy. Only agents targeting *Proteus* spp. are shown. Abbreviations/acronyms used in the table are as follows: Pat. no. – patient number; PoD – postoperative day; rTHA – revision total hip arthroplasty; rTKA – revision total knee arthroplasty; DAIR – debridement antibiotic, and implant retention; UPIC – unexpected, positive intraoperative culture; PIT – piperacillin–tazobactam; TRS – trimethoprim–sulfametrole; AMC – amoxicillin/clavulanic acid. Tier classifications are as follows: 1 – successful without suppressive antibiotic therapy; 3B – septic revision >1 year after PJI diagnosis; 3D – septic revision ≤1 year after PJI treatment; 3E – amputation, resection arthroplasty, and arthrodesis; 3F – retained spacer; 4A – death within 1 year of PJI treatment.

### 3.2 Resistance pattern and antimicrobial treatment

**Figure 1:**
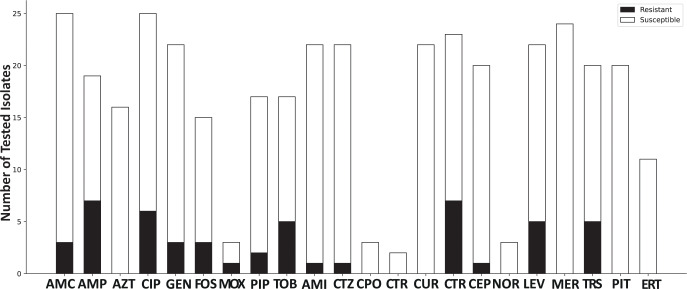
Antibiotic resistance pattern, giving the total number of tested isolates categorized as resistant and susceptible. Abbreviations used in the figure are as follows: AMC – amoxicillin/clavulanic acid; AMP – ampicillin; AZT – aztreonam; CIP – ciprofloxacin; GEN – gentamicin; FOS – fosfomycin; MOX – moxifloxacin; PIP – piperacillin; TOB – tobramycin; AMI – amikacin; CTZ – ceftazidime; CIX – cefixime; CPO – cefpodoxime; CTR – ceftriaxone; CUR – cefuroxime; CEP – cefepime; NOR – norfloxacin; LEV – levofloxacin; MER – meropenem; TRS – trimethoprim–sulfamethoxazole; PIT – piperacillin–tazobactam; ERT – ertapenem.

Antibiograms showed resistance against amoxicillin clavulanic acid (in 3 of 25 cases, 12 %), ampicillin (in 7 of 19 cases, 36.8 %), ciprofloxacin (in 5 of 25 cases, 20 %), gentamicin (in 3 of 22 cases, 13.6 %), fosfomycin (in 3 of 15 cases, 20 %), piperacillin (in 2 of 17 cases, 11.8 %), tobramycin (in 5 of 17 cases, 29.4 %), amikacin (in 1 of 22 cases, 4.5 %), ceftazidime (in 1 of 22 cases, 4.5 %), cefuroxime (in 7 of 23 cases, 30.4 %), cefepime (in 1 of 20 cases, 5 %), and trimethoprim–sulfamethoxazole (in 5 of 20 cases, 25 %). With respect to fluoroquinolones, ciprofloxacin showed resistance in 5 out of 25 tested samples (20 %), moxifloxacin showed resistance in 1 out of 3 tested samples (33.3 %), and levofloxacin showed resistance in 5 out of 22 tested samples (22.7 %). Antibiotic resistance patterns are shown in Fig. 1.

Perioperatively, cefuroxime alone or in combination with other antibiotics was administered in 7 out of 26 patients. Teicoplanin was given to 9 out of 26 patients during surgery and had already been started in 6 out of 9 patients preoperatively because of previous GP infections in some cases and continued during surgery. The remaining patients received various other antibiotics as empirical therapy. Detailed antibiotic treatments against *Proteus* spp. are presented in Table 3, and antibiotic therapies targeting other pathogens are summarized in Table S1 in the Supplement. Empiric therapy was then de-escalated according to the resistance pattern. The antimicrobial treatment was given for a mean of 14.4 ± 7.5 weeks. A fluoroquinolone was administered in 18 out of 26 cases, with ciprofloxacin used in 10 cases (36 %) and moxifloxacin in 6 cases (23.1 %). In 10 of 26 cases (36 %), ciprofloxacin alone or combined with other antibiotics was given. Ciprofloxacin administration showed a significant association with a success rate of 5 out of 10 cases (50 %; p=0.018 ). However, no statistically significant association between all fluoroquinolones and the success rate could be identified ( p=0.292 ).

### 3.3 Surgical procedures and outcomes

**Figure 2:**
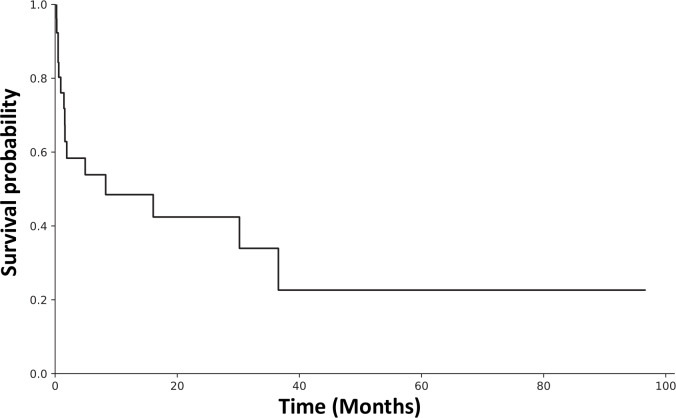
Kaplan–Meier estimates of the probability of infection-free survival after *Proteus* spp. culture-positive PJI in rTHA and rTKA for 25 patients.

Overall, in the 26 patients, 9 (34.6 %) infected primary TJA and 17 (65.4 %) infected revision TJAs were identified. Infected revision TJAs were primarily associated with persistent infections in 14 of 17 cases (82.4 %), with other microorganisms identified in prior septic revisions. In the case of chronic infections of revision TJAs, a higher failure rate was observed ( p=0.041 ) compared to acute infections. Polymicrobial and monomicrobial infections did not significantly correlate with the success or failure rate ( p=0.612 ). Revisions performed at culture sampling were debridement antibiotic, and implant retention (DAIR) in 10 of 26 cases (38.5 %); two-stage revisions in 12 of 26 cases (46.2 %; spacer in 9 of the 12 cases and resection arthroplasty in 3 of the 12 cases); septic single-stage revision in 3 of 26 cases (11.5 %); and presumed aseptic single-stage revision in 1 of 26 cases (3.8 %). Differences in success rates depending on the surgical procedure were not observed ( p=0.579 ), with overall success in 6 of 26 cases (23.1 %). The highest success rate of 1 of 3 cases (33.3 %) was observed in single-stage procedures, whereas the success rate in two-stage procedures was 3 of 12 cases (25.0 %). In contrast, DAIR procedures were related to septic failure in 8 of 10 cases (80.0 %). Additionally, the UPIC case resulted in a septic failure in the consecutive re-revision procedure. The overall reinfection rate is illustrated in Fig. 2. Infection-free survival after 12 months was 48.5 %, whereas it was 22.6 % after 40 months.

## 4 Discussion

In this study, *Proteus* pathogen was identified in 26 of 1776 cases (1.5 %) in rTHAs and rTKAs. Notably, *Proteus*-species-associated PJIs were observed to be more prevalent in rTHAs (88.5 %) compared to rTKAs (11.5 %). Furthermore, the majority of the *Proteus*-species-associated PJIs were identified in polymicrobial infections (15 of 26 cases, 57.7 %) and chronic revisions (16 of 25 cases, 64 %), resulting in a high failure rate.

The prevalence of *Proteus* spp. in 1.5 % of rTHA and rTKA surgeries observed in our study was lower compared with the respective values of 4.8 % and 2.6 % reported by Benito et al. (2016) and van Veghel et al. (2024). Moreover, the higher incidence of *Proteus*-species-associated PJI in rTHA (88.5 %) than in rTKA (11.5 %) is corroborated by van Veghel et al. (2024). Polymicrobial infections were more prevalent in chronic rTHA ( p=0.02 ) than in chronic rTKA ( p=0.667 ), with *Enterococcus* spp., *Pseudomonas* spp., and *S. epidermidis* being the most frequent pathogens. *Enterococcus *spp. and *E. coli* as co-pathogens were especially prevalent in rTHA, mainly due to the hip's unique anatomical and microbiological environment and gut colonization of *Enterococcus* spp., which could lead to contamination during or after surgery (Chisari et al., 2022; Mitterer et al., 2024). The reason for the more frequent association of *Proteus* with rTHA compared to rTKA remains unknown. However, Loewik et al. (2019) demonstrated a significantly higher prevalence of *Proteus* spp. in rTHA in extremely obese patients compared to rTKA, which is consistent with our findings. Although the association of obesity and *Proteus* spp. infections was not investigated in our cohort, compared to other cohorts, the mean BMI in our cohort was >30 kg m^−2^, which could have influenced the risk of *Proteus* infections. While *Proteus* is known for its common colonization of the urogenital tract and its association with UTIs, recent studies have identified a correlation between UTIs and PJIs (Blanchard et al., 2022). However, the present study could not identify any association between UTIs and *Proteus*-species-associated PJIs.

Notably, persistent infections with *Proteus* spp. were rare, as only one patient presented with a *Proteus* infection in two consecutive surgeries. This may be attributed to the frequent changes in microorganisms throughout revision surgeries and their association with polymicrobial infections, making their detection more difficult (Frank et al., 2021; McCulloch et al., 2023). As previous studies have shown, changes in the microbial spectrum throughout revision surgeries are not necessarily considered to be new infections but, rather, infections that have not been previously detected (Frank et al., 2021). This study showed that polymicrobial infections were especially found in chronic revisions, as corroborated by previous findings (Li et al., 2021). Additionally, studies found that the number of pathogens increases with the number of revisions, leading to a higher occurrence of polymicrobial infections in chronic PJI (McCulloch et al., 2023). Kavolus et al. (2019) recently proved that polymicrobial infections have poorer outcomes. In congruence with their findings, we also observed a low success rate of *Proteus*-species-associated polymicrobial PJIs.

The most common antibiotic treatment in our study included fluoroquinolones, a recommended treatment for *Proteus* spp. infections (Osmon et al., 2012) due to their biofilm penetration (Przekwas et al., 2022) and ability to achieve effective therapeutic concentrations in tissue as well as bone penetration (Landersdorfer et al., 2009). This was evident, as PJIs treated with ciprofloxacin had a higher success rate. Previous studies have also shown a better outcome in patients with GN PJI treated with ciprofloxacin (Martínez-Pastor et al., 2009). However, *Proteus* isolates in our study showed resistance to ciprofloxacin in 20 % of the tested samples, resulting in treatment failures. Kwiecinska-Piróg et al. (2013) reported even higher resistance of *Proteus* spp. isolates to ciprofloxacin (40 %, or 20 of 50 cases) (2013).

Two-stage procedures are recommended for GN and chronic PJI (Hsieh et al., 2009; Kildow et al., 2022). In this study, most interventions were two-stage revisions due to the chronic nature of the PJIs. Two-stage and one-stage revisions had the highest success rate, up to one-third of all cases, resulting in infection control. The low number of DAIR procedures is because most of the *Proteus*-species-associated PJIs were chronic PJIs. Previous studies showed that DAIR procedures are not recommended in chronic revision cases or GN-associated infections (Zhu et al., 2021). However, due to the small number of surgical procedures, no recommendation for a surgical procedure can be made.

This study has limitations due to its retrospective nature and the small number of *Proteus*-species-associated PJIs identified, resulting in a heterogeneous analysis. Moreover, the number of sample acquisitions differed greatly depending on the surgeon's preference, and tissue extraction was not standardized earlier. Consequently, many swabs were accepted as intraoperative cultures, although they were not tissue cultures. This discrepancy may result in differences and inaccuracies in microbial analysis. Furthermore, this work was a single-centre study, resulting in differences in PJI etiology and antibiotic resistance compared to other institutions. However, to our knowledge, no prior studies have focused on *Proteus*-species-associated PJIs in this context, making direct comparisons challenging.

## 5 Conclusion

In conclusion, *Proteus* spp. present significant challenges in revision arthroplasty, as they mainly occur in polymicrobial and chronic revision PJIs, with a higher prevalence in hip PJIs. Treatment options for chronic and polymicrobial infections are limited and make it difficult to carry out successful treatment. Although fluoroquinolones, especially ciprofloxacin, showed a promising antimicrobial treatment, the growing resistance is concerning. Future studies are required to develop pathogen-specific strategies for optimal treatment of these cases.

## Supplement

10.5194/jbji-10-265-2025-supplement The supplement related to this article is available online at https://doi.org/10.5194/jbji-10-265-2025-supplement.

## Supplement

10.5194/jbji-10-265-2025-supplement
10.5194/jbji-10-265-2025-supplement The supplement related to this article is available online at https://doi.org/10.5194/jbji-10-265-2025-supplement.

## Data Availability

Data can be provided upon reasonable request.

## Appendix

The supplement related to this article is available online at https://doi.org/10.5194/jbji-10-265-2025-supplement.
