# Supplementary material for: Proteus-species-associated periprosthetic hip and knee joint infections – a 15-year cohort analysis
Source: J Bone Jt Infect. 2025 Aug 11;10(4):265–75. doi: 10.5194/jbji-10-265-2025 (PMC12345592; doi:10.5194/jbji-10-265-2025)
Supplement: The supplement related to this article is available online at https://doi.org/10.5194/jbji-10-265-2025-supplement. [file jbji-10-265-2025-supplement.pdf]

Supplement of J. Bone Joint Infect., 10, 265–275, 2025
https://doi.org/10.5194/jbji-10-265-2025-supplement
© Author(s) 2025. CC BY 4.0 License.

Supplement of

***Proteus*-species-associated periprosthetic hip and knee joint infections – a 15-year cohort analysis**

Veronika Achatz et al.

Correspondence to: Jochen G. Hofstaetter (researchlab@oss.at)

The copyright of individual parts of the supplement might differ from the article licence.

Pat.	Joint	Surgical procedure	Microorganisms	Prior infections	Initial antibiotic therapy	Targeted antibiotic therapy	Targeted therapy initiation (PoD)	Mixed infection – targeted AB ****	Duration (weeks)	Tier class.	success /failure
2	rTHA	Two-stage	*P. mirabilis+S. epidermidis*	*P. mirabilis*	Teicoplanin**+Moxifloxacin**	**Ceftriaxone+Ciprofloxacin**	4	**TRS+Ciprofloxacin**	7	4A	failure
3	rTHA	One-stage	*P. mirabilis+P. aeruginosa*	*Culture negative*	Cefuroxime*	**Meropenem**	4	**Meropenem**<br>**Ciprofloxacin**	8	1	success
5	rTHA	DAIR	*P. mirabilis+*<br>*S. lugdunensis+S. epidermidis*	-	Teicoplanin**	**Rifampicin**<br>Doxycyline<br>Ciprofloxacin	3<br>3<br>7	**Rifampicin**<br>Doxycyline<br>**Ciprofloxacin**<br>**Fusidic acid+Ciprofloxacin**	9	3B	failure
6	rTHA	Two-stage	*P. mirabilis+P. aeruginosa*	*S. aureus*	Teicoplanin***+Meropenem***	**Meropenem**	0	**Meropenem**<br>**Ciprofloxacin**	6	1	success
7	rTHA	DAIR	*P. mirabilis+S. lugdunensis*	-	Doxycycline*+Moxifloxacin***	**Rifampicin**<br>Clindamycin<br>TRS	1<br>1<br>8	Rifampicin<br>Clindamycin<br>**TRS**	6	1	success
9	rTHA	Two-stage	*P. mirabilis+*<br>*S. epidermidis+C. avidum*	-	Cefuroxime***+Doxycycline*	**Aztreonam**<br>Daptomycin	5<br>5	**Aztreonam**<br>Daptomycin<br>**Moxifloxacin**	8	3D	failure
16	rTHA	Two stage	*P. mirabilis+E. faecalis*	*unknown*	Cefuroxime***+Moxifloxacin***	**Moxifloxacin+**<br>Rifampicin	0<br>3	**Moxifloxacin**<br>Rifampicin<br>**AMC**	12	3F	failure
17	rTHA	DAIR	*P. mirabilis+S. aureus*	-	Clindamycin***	**Meropenem**	6	**Meropene+Moxifloxacin**<br>**Ciprofloxacin+**Clindamycin	12	3D	failure
18	rTKA	One-stage	*P. mirabilis+S. epidermidis*	-	Cefuroxime**+Doxycycline*	**Cefalexin**	11	**Cefalexin**<br>**TRS+** Rifampicin<br>**TRS+** Fusidic acid	12	4A	failure
19	rTHA	One-stage	*P. mirabilis+E. faecalis+*<br>*E. coli*	-	Cefazolin***	**Cefotaxime+**<br>Daptomycin	7<br>9	**Cefotaxime**<br>**Aztreonam**+Daptomycin<br>**Meropenem**<br>**Ceftazidime/Avibactam+Fosfomycin**<br>**Ceftazidime/Avibactam+Levofloxacin**	30	3D	failure
20	rTHA	Two stage	*P. mirabilis+E. faecalis*	-	PIT***	**Meropenem**	unknown	**Meropenem**<br>**Ciprofloxacin**<br>Dalbavancin	24	1	success
22	rTHA	DAIR	*P. vulgaris+E. faecalis*	*Culture negative*	Cefazolin**	**PIT**	7	**PIT, Moxifloxacin, AMC, TRS,**<br>**Levofloxacin,** Teicoplanin	17	4A	failure
23	rTHA	Two-stage	*P. mirabilis+E. coli*	*S. epidermidis*	Doxycycline**+Teicoplanin**	**Trimethoprim**	4	**Trimethoprim**<br>**Meropenem**	12	4A	failure
25	rTKA	Two-stage	*P. mirabilis+E. faecalis,*<br>*K. pneumoniae, C. koseri*	*unknown*	PIT***+Cefazolin*	**PIT+**<br>Teicoplanin	0<br>4	**PIT**<br>**Aztreonam**<br>**Ceftazidime**<br>**Ciprofloxacin**<br>Dalbavancin	12	3D	failure
26	rTKA	DAIR	*P. mirabilis+P. aeruginosa,*<br>*C. koseri, E. coli*	*C. glabrata+*<br>*S. epidermidis*	Cefuroxime*	**Meropenem**	0	**Meropenem**	8	3E	

Table S1: Detailed microbial profiles, surgical details, antibiotic therapy, and success/failure rate as per the Tier classification in patients with mixed infections. Antibiotics targeted for *Proteus* spp. are indicated in bold, while those targeting co-pathogens are written in regular fonts. Every line represents one patient. Patients 23 and 24 refer to the same patient but are mentioned separately because they underwent different surgeries and treatments. Antibiotic agents targeting co-pathogens are not mentioned in this table. Please see the supplementary material for additional details on antibiotic therapies in polymicrobial infections.

*Antibiotic therapy was given as perioperative prophylaxis; **antibiotic therapy was started before surgery and continued during surgery; ***Empirical antibiotic therapy, which was started during surgery and changed accordingly to antibiogram; ****AB changes classified as continued, switched, de-/escalated, or salvage therapy. Pat., patient number; PoD postoperative day; rTHA, revision total hip arthroplasty; rTKA, revision total knee arthroplasty; DAIR, debridement antibiotic and implant retention; UPIC Unexpected, positive intraoperative culture; PIT Piperacillin-tazobactam, TRS trimethoprim-sulfametrol, AMC amoxicillin/clavulanic acid; Tier classification: 1 successful without suppressive antibiotic therapy, 3B septic revision > 1year after PJI diagnosis; 3D septic revision ≤ 1 year after PJI treatment; 3E amputation, resection, arthroplasty, arthrodesis; 3F retained spacer; 4A death within 1 year of PJI treatment.
